# Evaluation of the Diagnostic Accuracy of the Quantitative Point‐of‐Care SD Biosensor Standard G6PD Test for Assessment of G6PD Deficiency in Infectious Diseases

**DOI:** 10.1111/ijlh.70030

**Published:** 2025-11-23

**Authors:** Flavia Regina Medeiros da Silva, Alexandre Gomes Vizzoni, Daniela Mendes‐de‐Almeida, Joanna Bokel, Raquel de Vasconcellos Carvalhaes de Oliveira, André Machado Siqueira, Yasmine Rangel Vieira, Simone da Costa Cruz Silva

**Affiliations:** ^1^ Hematology and Hemotherapy Service, Evandro Chagas National Institute of Infectious Diseases (INI) Oswaldo Cruz Foundation Rio de Janeiro Brazil; ^2^ Clinical Epidemiology Laboratory Evandro Chagas National Institute of Infectious Diseases (INI), Oswaldo Cruz Foundation Rio de Janeiro Brazil; ^3^ Acute Febrile Illnesses Laboratory Evandro Chagas National Institute of Infectious Diseases (INI), Oswaldo Cruz Foundation Rio de Janeiro Brazil; ^4^ Immunobiological Technology Institute, Bio‐Manguinhos, Oswaldo Cruz Foundation Rio de Janeiro Brazil; ^5^ Molecular Diagnosis and Cytometry Service Evandro Chagas National Institute of Infectious Diseases (INI), Oswaldo Cruz Foundation Rio de Janeiro Brazil

**Keywords:** diagnostic accuracy, glucose‐6‐phosphate dehydrogenase deficiency, hemolysis, infectious diseases, rapid diagnostic test

## Abstract

**Background:**

G6PD deficiency affects about 500 million people worldwide and is prevalent in many malaria‐endemic settings. People with G6PD deficiency are at risk of hemolysis when exposed to certain medications, including 8‐aminoquinoline drugs used to treat Plasmodium vivax malaria. Increasing access to testing for G6PD deficiency at or near the point of care is critical to expanding the safe treatment of *P. vivax* malaria. We aimed to evaluate the performance of a semiquantitative test for G6PD deficiency, the SD Biosensor Standard G6PD Test with the Brewer's method, in a reference site for the treatment of infectious diseases.

**Method:**

We evaluate the diagnostic accuracy performance of the SD Biosensor Standard G6PD Test in 125 individuals with infectious diseases and other illnesses.

**Main Results:**

We observed a trend of concordance between the G6PD status (Deficient or Normal), with low frequencies of discordances in the screenings. The strength of agreement between G6PD tests was classified as almost perfect in all participants (*k* = 0.82, 95% CI = 0.66, 0.97) and in sex subgroups: females (*k* = 0.83, 95% CI = 0.59, 1.00) and males (*k* = 0.81, 95% CI = 0.59, 1.00). The total concordance percentage (number of concordances/total) was 95% for females, 97% for males, and 96% overall.

**Conclusion:**

The SD Biosensor STANDARD G6PD test is an innovative point‐of‐care solution. It offers quantitative measurement of glucose‐6‐phosphate dehydrogenase activity while normalizing for hemoglobin levels. This advancement enables its use in lower‐tier clinical and laboratory settings, expanding access to accurate G6PD testing.

## 1 Introduction

Glucose‐6‐phosphate dehydrogenase (G6PD) deficiency is an X‐linked, hereditary condition caused by mutations in the G6PD gene, which will result in protein variants with different enzyme activity levels. G6PD provides reducing power to NADPH (reduced form of nicotinamide adenine dinucleotide phosphate) [1]. G6PD is ubiquitously expressed in all cells and is essential to counterbalance oxidative stress triggered by several oxidant agents. NADPH's effect is critical in red blood cells due to the lack of nucleus [1, 2].

G6PD deficiency is associated with multiple episodes of biochemical and clinical abnormalities, including fatigue, back or abdominal pain, anemia, jaundice, splenomegaly, gallstones, fever, increased unconjugated bilirubin, lactate dehydrogenase, and reticulocytosis [1, 2]. A wide range of G6PD deficiency clinical phenotypes can be observed. Males who are hemizygous for the G6PD gene can have regular gene expression or be G6PD‐deficient. Females, who have two copies of the X chromosome, can have regular gene expression, be heterozygous, or be homozygous deficient. Nevertheless, most individuals are asymptomatic. Clinical manifestations are usually mild; however, some individuals may develop acute and sometimes very severe hemolytic anemia when this is triggered by ingestion of fava beans, or by any of a number of drugs (e.g., primaquine, rasburicase), or more rarely by infection.

G6PD deficiency is a widespread genetic trait and the most common human enzyme defect. It is present in more than 500 million people worldwide, with prevalence ranging from nearly 0% in Native American populations to up to 20% in populations of African and Asian ancestry [2]. The global overlapping distribution of malaria and mutated G6PD alleles suggests the hypothesis that G6PD deficiency is protective against malaria. A plausible protection mechanism is the early elimination of infected red blood cells by macrophages [3]. Malaria remains a serious public health problem in Brazil, affecting isolated communities, such as indigenous groups [4]. Severe G6PD deficiency is uncommon across the Amazon Basin but severe haemolysis has been occasionally reported after malaria treatment with primaquine [5]. In Brazil, G6PD deficiency prevalence is approximately 5%, but can reach up to 12.9% in some populations [6, 7].

The gold standard for the measurement of G6PD activity is a quantitative Ultraviolet (UV) spectrophotometric assay [8, 9]. Nevertheless, this test is complex and requires laboratory infrastructure and experienced personnel [10]. The methemoglobin reduction test (Brewer's method) is a qualitative spot test most used for G6PD deficiency screening since it is cheaper and easier to perform. However, it is imprecise particularly in females, requires a cold chain, some laboratory infrastructure, trained personnel, and is time‐costing. Point‐of‐care (POC) tests are simple semi‐quantitative tests that are becoming increasingly important in health care for out‐of‐reach communities to increase safe access to infectious disease treatment. They measure G6PD enzymatic activity, and total hemoglobin concentration in fresh blood, and could be performed and interpreted by health workers at the bedside or in the field in less than 30 min [8]. The aim of this study is to measure diagnostic accuracy between Brewer's method and POC for G6PD deficiency diagnosis in patients with infectious diseases.

## 2 Materials and Methods

A cross‐sectional diagnostic accuracy study to evaluate concordance analysis between Brewer and POC Standard G6PD in individuals older than 18 years old enrolled between May 2020 and December 2021 at Evandro Chagas National Institute of Infectious Diseases, Oswaldo Cruz Foundation (FIOCRUZ), Rio de Janeiro, Brazil. Clinical, demographic, and laboratory features were obtained from the patients' medical records. The diagnostic tests were performed from venous specimens using Brewer method as reference assay.

Due to technical device limitations, hemoglobin concentrations less than 7.0 g/dL were exclusion criteria.

The sample size calculation was 125 individuals and performed using the WinPepi program, version 11.63 [11], with a confidence interval (CI) of 0.95, standard deviation (SD) of 0.05, and an estimated prevalence of G6PD of 0.08. The individuals were sequentially recruited during medical visits.

To collect the blood samples, we used a standard venipuncture kit and collected 4 mL of venous blood from each participant, which was deposited in a tube treated with K2EDTA anticoagulant. The samples were stored at 4°C for a maximum of 4 h between collection time and laboratory analysis of G6PD activity.

The two individuals responsible for executing and reviewing the index and reference standard tests had undergone training, possessed experience in evaluating the tests, and were aware of the results from the other test, thus were not blinded to them.

The index test and reference standard were conducted at the same time, with no treatment administered in between.

### 2.1 Methemoglobin Reduction Test (Brewer Method)

To 200 μL of whole blood from the EDTA tube, 10 μL of sodium nitrite‐glucose and 10 μL of sodium nitrite solution were added to the methylene blue solution. After incubation in a water bath at 37°C for 3 h, this solution was transferred to a tube containing 10 mL of type 2 water (deionized water to remove contaminants and achieve high levels of purity) and mixed by inversion. By visual reading, the color formed in the sample tube was compared with the colors of the positive and negative control tubes. For normal G6PD activity, the final reaction should be bright red, as the nitrite solution converts hemoglobin to methemoglobin, which is reconverted to hemoglobin in the presence of methylene blue. In the presence of reduced G6PD activity, the final color is brown as methemoglobin formation occurs without reconversion [12].

### 2.2 Biosensor Standard G6PD Test (Point of Care)

For testing with venous specimens, 10 μL of venous whole blood was transferred into the test's buffer tube using a professional pipette. The specimen was then mixed with the buffer 10 to 15 times using the pipette, and 10 μL of the mixed specimen was transferred onto the device's test strip using a new pipette tip. Results were obtained within 2 min. The test reports results in U/g Hb for G6PD activity and g/dL for hemoglobin concentration [10, 13].

The manufacturer of the standard G6PD Test (SD Biosensor) has established single thresholds for G6PD activity (U/g Hb) for the test's classification of G6PD status on venous specimens based on previously published clinical data [10, 14, 15]. The thresholds recommended by the manufacturer are: ≤ 3.9 U/g Hb for G6PD‐deficient males and females (≤ 30% activity), 4.0 to 6.0 U/g Hb for G6PD‐intermediate females (> 30% to ≤ 70% activity), ≥ 4.0 U/g Hb for G6PD‐normal males (> 30% activity), and > 6.0 U/g Hb for G6PD‐normal females (> 70% activity).

According to the current World Health Organization (WHO) case definition, males and females with G6PD activity levels of less than 30% of the mean standard value are considered to be G6PD deficient; heterozygous females with activity levels between 30% and 80% are considered to be G6PD intermediate; males with > 30% activity levels and females with > 80% activity levels are deemed to be G6PD normal [16].

### 2.3 Statistical Analysis

Statistical analyses were performed using the R Language for Statistical Computing version 4.2.1 (R Core Team, Vienna, Austria). Categorical data were presented as frequencies (counts and percentages). Normality of continuous data was rejected by Shapiro–Wilk's test, and we used nonparametric tests. The Mann–Whitney test was used to compare the distribution of G6PD enzyme activities between males and females. Fisher's exact test was used to verify the association between the qualitative variables and gender.

To evaluate the concordance between tests we provided the frequencies on the G6PD status between Biosensor and Brewer's method, Cohen's kappa index (*k*), and total concordance percentage (ratio between the number of concordances and total).

The kappa coefficient is calculated by comparing the observed agreement (the proportion of ratings on which the raters agree) with the expected agreement (the proportion of ratings that would be expected to agree by chance). The kappa coefficient can range from 0 to 1. A value of 1 indicates perfect agreement, 0 indicates agreement equal to chance. Generally, values above 0.6 are considered to indicate substantial agreement, while values below 0.4 suggest poor agreement [17].

The concordance estimates were also stratified by sex and race. Intermediate was assumed to be deficient in the comparison between all participants.

All statistical tests were two‐sided, and a *p*‐value < 0.05 was considered statistically significant. However, due to critical issues with the exclusive use of *p*‐values in the decision analysis [18], we provided confidence intervals (95% CI).

## 3 Results

A total of 125 subjects were recruited for the study, 38 females and 87 males. The median age was 42.0 years (range 18–76 years) in females and 45.0 years (range 19–78 years) in males. In relation to race, the majority of participants were black/mixed (*n* = 89, 71.2%). The proportion of participants with 6–9 years of education (*n* = 67, 53.6%) was the highest, followed by ≥ 10 years (42, 33.6%) and < 6 years (1, 0.8%). The most common diagnosis was human immunodeficiency virus (HIV) in 39.5%, followed by malaria (13.2%) and mycosis (13.2%). We did not observe differences in the characteristics according to sex (Table 1).

**TABLE 1:** Demographic and clinical characteristics of the study population.

Variable	Categories	Female		Male		*p*
		(*N* = 38)		(*N* = 87)		
		*n* (%)	95% CI	*n* (%)	95% CI	
Age groups	≤ 29 years	10 (26.3)	13.4–43.1	13 (14.9)	8.2–24.1	0.515
	30–39 years	6 (15.8)	6.0–31.2	21 (24.1)	15.6–34.5	
	40–49 years	8 (21.1)	9.5–37.3	24 (27.6)	18.5–38.2	
	50–59 years	7 (18.4)	7.7–34.3	14 (16.1)	9.0–25.5	
	≥ 60 years	7 (18.4)	7.7–34.3	15 (17.2)	9.9–26.8	
Race	White	10 (26.3)	14.0–43.4	26 (29.9)	20.8–40.8	0.831
	Black/mixed	28 (73.7)	56.6–86.0	61 (70.1)	59.2–79.2	
Anemia status^a^	Non	10 (26.3)	13.4–43.1	29 (33.3)	23.5–44.2	0.419
	Mild	9 (23.7)	11.4–40.2	22 (25.3)	16.7–35.7	
	Moderate	8 (21.1)	9.5–37.3	22 (25.3)	16.7–35.7	
	Severe	11 (28.9)	15.4–45.9	14 (16.1)	9.0–25.5	
Disease diagnosis	HIV	15 (39.5)	24.0–56.6	31 (35.6)	25.6–46.6	0.093
	Tuberculosis	1 (2.6)	0.0–13.1	7 (8.0)	3.2–15.8	
	Mycosis^b^	5 (13.2)	4.4–28.0	17 (19.5)	11.8–29.4	
	Malaria	5 (13.2)	4.4–28.0	21 (24.1)	15.6–34.5	
	Other infectious diseases^c^	4 (10.5)	2.9–24.8	2 (2.3)	2.0–8.0	
	Noninfectious diseases^d^	8 (21.1)	9.5–37.3	9 (10.3)	4.8–18.7	

Abbreviations: 95% CI, 95% confidence intervals; G6PD, glucose‐6‐phosphate dehydrogenase; HIV, human immunodeficiency virus.

^a^ Anemia status following published clinically relevant hemoglobin concentration thresholds from the World Health Organization (WHO).

^b^ Mycosis aspergillosis, paracoccidioidomycosis, histoplasmosis and sporotrichosis.

^c^ Other infectious diseases human T‐cell leukemia virus type 1, Chagas disease, leprosy, toxoplasmosis and COVID‐19.

^d^ Rheumatoid arthritis, systemic lupus erythematosus, anemia hemolytic autoimmune and sickle cell disease. #T‐test was not calculated due to count = 0.

**FIGURE 1:**
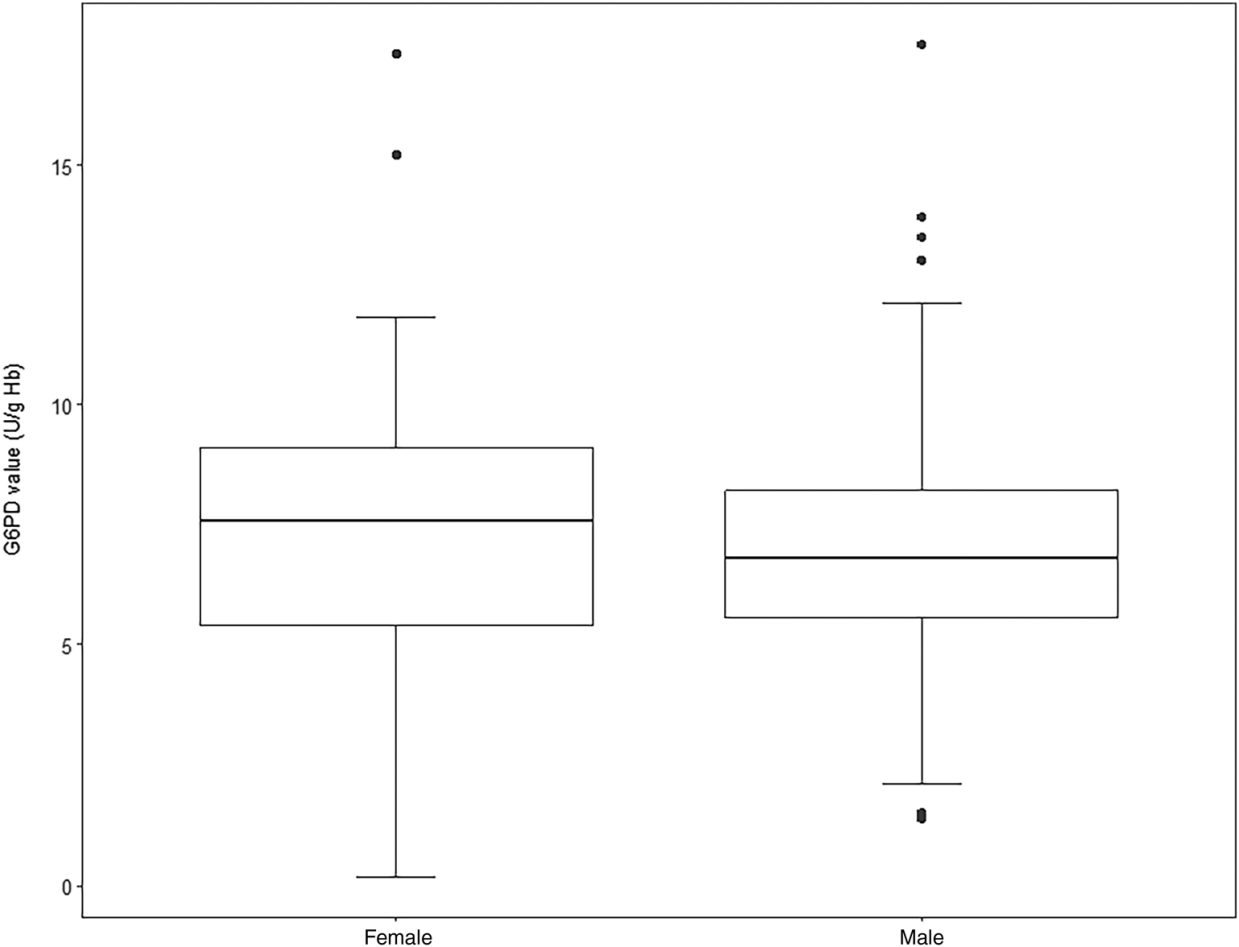
Distribution of G6PD enzyme activities according to sex in an infectious disease hospital located in a central area of Rio de Janeiro, Brazil.

The median of G6PD activity was 7.1 U/gHb (minimum = 0.2 and maximum = 17.5). Moreover, we did not observe a difference in the G6PD enzyme activities between males and females, as illustrated in Figure 1 (Mann–Whitney test *p* = 0.313).

The concordance analysis between Biosensor and Brewer's method is shown in Table 2. We observed a trend of concordance between the G6PD status (Deficient or Normal), with low frequencies of discordances in the screenings. The strength of agreement between G6PD tests was classified as almost perfect in all participants (*k* = 0.82, 95% CI = 0.66, 0.97) and in sex subgroups: females (*k* = 0.83, 95% CI = 0.59, 1.00) and males (*k* = 0.80, 95% CI = 0.59, 1.00). The total concordance percentage (number of concordances/total) was 95% for females, 97% for males, and 96% overall.

**TABLE 2:** Comparison between Biosensor Standard G6PD test performance and Reference Brewer test for screening G6PD deficiency.

			G6PD Brewer test		Total	Kappa
			Normal	Deficient		
Sex						
Female	Biosensor Standard^a^ G6PD test	Normal	30	0	30	0.83
		Deficient	2	6	8	
Male	Biosensor Standard G6PD test	Normal	77	1	78	0.80
		Deficient	2	7	9	
Skin color/Race						
White	Biosensor Standard G6PD test	Normal	28	0	28	0.92
		Deficient	1	7	8	
Black/Mixed	Biosensor Standard G6PD test	Normal	79	1	80	0.73
		Deficient	3	6	9	
All participants	Biosensor Standard G6PD test	Normal	107	1	108	0.82
		Deficient	4	13	17	
	Total		111	14	125	

*Note:* Assay specifications: The threshold of G6PD Biosensor Standard for identifying subjects as deficient is less than 3.9 IU/g Hb, intermediate is 4.0 to 6.0 IU/g Hb (females) and normal is more than 6.0 IU/g Hb (females) and 4.0 IU/g Hb (male).

^a^ Intermediate was assumed to be Deficient in the comparison between all participants.

The sensitivity of the Biosensor Standard G6PD Test compared to the Brewer method was 92.8%. The specificity was 96.4%. The positive predictive value was 76.5% and the negative predictive value was 99%.

## 4 Discussion

Our results show that the POC Biosensor method presents a strong agreement with the test Brewer's. This finding is of great value in the infectious diseases setting, where both diseases and treatment could induce acute hemolysis and may require prompt conduct.

Although Brewer's test indicated four deficient cases as normal, reports in the literature have demonstrated that this method has limitations in patients with elevated bilirubin. Such discrepancies may result from bilirubin interfering with methemoglobin reduction, from different mechanisms of oxidative stress, or simply from bilirubin's color interfering with the subjective interpretation of the test's color change [12, 19].

Antimalarials, sulfonamides, sulfones, antibiotics, antipyretics, and other drugs for the treatment of these diseases are associated with drug‐induced hemolytic anemia in G6PD deficient patients. Also, hepatitis viruses A and B, cytomegalovirus, pneumonia, and typhoid fever infection could trigger hemolysis [1, 20].

The range of clinical conditions among patients undergoing G6PD testing reflects the care profile of INI, located in Rio de Janeiro. HIV infection was the most frequent diagnosis, followed by mycoses, malaria, and tuberculosis. Other infectious diseases included human T‐cell leukemia virus type 1, Chagas disease, leprosy, toxoplasmosis, and COVID‐19. In Brazil, G6PD testing shows marked regional variation in both prevalence and clinical screening practices, largely influenced by malaria epidemiology and the associated risk of drug‐induced hemolysis. Of particular interest is the Brazilian Amazon, where malaria remains endemic [21].

Heterozygous females are genetic mosaics because of the epigenetic event X‐chromosome inactivation, leading to random inactivation of the X chromosome in different cells. Eventually, the abnormal cells of a heterozygous female can be as deficient for G6PD as those of a G6PD‐deficient male [1]. Although heterozygous women, on average, have less severe clinical manifestations than G6PD‐deficient males, some develop severe acute hemolytic anemia [1].

Diagnosing G6PD can be challenging in an acute hemolysis crisis due to false‐negative results. If G6PD deficiency is suspected, but the screening test shows normal enzyme activity, it should be repeated 2–3 months after the acute hemolytic episode [8]. In parallel, a blood film examination shows typical findings of oxidant damage within 2 days from the clinical onset. Although molecular analysis is the only method for a definitive diagnosis in females, it is not broadly available and does not reflect protein expression. Indeed, UV *spectrophotometry* and genotyping of G6PD variants were not available in this study.

One of the strengths of this work is that it highlights that the POC Biosensor Standard G6PD test is effective for screening G6PD enzyme‐deficient patients and has a low rate of false positives. Of note, the POC G6PD test is able to detect heterozygous individuals (intermediate), meaning those who carry a single altered copy of the G6PD gene. For many years, these heterozygote patients were not considered at risk due to the presence of a functional copy of the gene. However, heterozygotes can also be at risk of hemolytic reactions under certain conditions, such as the use of oxidizing medications or exposure to infections [20].

Finally, our results endorse the use of a simpler and faster method to diagnose G6PD deficiency. POC testing modalities have been evaluated in urban centers like Manaus and Porto Velho. These tests demonstrate high sensitivity and specificity for detecting both severe (< 30% activity) and intermediate (< 70% activity) deficiency [10]. POC testing is a rapid and cost‐effective strategy for assessment of G6PD status, with value in out‐of‐reach communities, avoiding hospitalization [22]. The incorporation of this new methodology potentially can improve healthcare and prevent iatrogenic measures induced by severe hemolysis, helping future decision‐making policies related to infectious diseases management.

The limitations of this study relate primarily to the sampling approach and the generalizability of the findings. The quantitative methodology employed may have constraints when applied to the chosen sampling strategy. In addition, the representativeness of participants across infectious disease case management settings is limited, and the study sample may not adequately capture the diversity of potential end users of POC G6PD tests across different health systems worldwide. Another limitation is that the comparison of POC G6PD results was made using the Brewer test as a reference, rather than a gold standard quantitative UV assay. Consequently, it is not possible to determine which method provided the correct result in cases of discrepancy.

There is a tendency to limit G6PD deficiency testing to patients from ethnic groups where the deficiency is common, such as those of African ancestry. However, in Brazil, inferring ethnic groups based on appearance or background can be inaccurate due to the high prevalence of mixed or partially known ancestries.

Therefore, we recommend conducting G6PD testing before administering high‐risk drugs. The most effective management strategy for G6PD deficiency is to prevent hemolysis by avoiding oxidative stressors.

## 5 Conclusion

Emerging quantitative point‐of‐care technologies are being developed to address two critical needs: the provision of rapid results to mitigate the risk of drug‐induced hemolysis (e.g., the use of primaquine for malaria treatment), and the ability to deliver reliable G6PD deficiency diagnostics in resource‐limited settings lacking access to centralized screening programs.

Given the potential severity of clinical manifestations including acute and occasionally fatal hemolytic anemia—associated with G6PD deficiency, particularly in the presence of oxidative stressors, early detection and preventive counseling are essential components of patient care. The established association between G6PD deficiency and jaundice further reinforces the need for proactive clinical strategies. Integrating enzyme activity assays with molecular diagnostic methods will increase the accuracy of confirmatory testing, providing clinicians with more conclusive data to guide appropriate diagnosis, treatment, and long‐term follow‐up.

## Author Contributions

Conceptualization: Alexandre Gomes Vizzoni, Flavia Regina Medeiros da Silva, and Simone da Costa Cruz Silva. Methodology: Alexandre Gomes Vizzoni, Yasmine Rangel Vieira, and Simone da Costa Cruz Silva. Formal analysis: Raquel de Vasconcellos Carvalhaes de Oliveira. Investigation: Flavia Regina Medeiros da Silva and Alexandre Gomes Vizzoni. Data curation: Alexandre Gomes Vizzoni, Flavia Regina Medeiros da Silva, and Raquel de Vasconcellos Carvalhaes de Oliveira. Writing – original draft preparation: Daniela Mendes‐de‐Almeida, Joanna Bokel, André Machado Siqueira, and Yasmine Rangel Vieira. Writing – review and editing: Daniela Mendes‐de‐Almeida, André Machado Siqueira, and Alexandre Gomes Vizzoni. Supervision: Alexandre Gomes Vizzoni, Daniela Mendes‐de‐Almeida, and Simone da Costa Cruz Silva. Project administration: Alexandre Gomes Vizzoni. All authors have read and agreed to the published version of the manuscript.

## Funding

The authors have nothing to report.

## Ethics Statement

This study was approved by the Research Ethics Committee of the Evandro Chagas National Institute of Infectious Diseases under number 04867118.8.0000.5262. All procedures followed regulatory guidelines and standards for research involving human beings as stated in Brazilian National Health Council Resolution 466/2012 and were conducted according to the principles expressed in the Declaration of Helsinki in order to safeguard the rights and welfare of the participants.

## Consent

Informed consent was obtained from all subjects involved in the study.

## Conflicts of Interest

The authors declare no conflicts of interest.

## Data Availability

The data that support the findings of this study are available from the corresponding author upon reasonable request.
